# Smoking, Alcohol Drinking and Esophageal Cancer: Findings from the JACC Study

**DOI:** 10.2188/jea.15.S212

**Published:** 2005-08-18

**Authors:** Kiyomi Sakata, Yoshiharu Hoshiyama, Seiji Morioka, Tsutomu Hashimoto, Tatsuya Takeshita, Akiko Tamakoshi

**Affiliations:** 1 Department of Hygiene and Preventive Medicine, Iwate Medical University School of Medicine.; 2 University of Human Arts and Sciences.; 3 Department of Public Health, Wakayama Medical University.; 4 Department of Preventive Medicine/ Biostatistics and Medical Decision Making, Nagoya University Graduate School of Medicine.

**Keywords:** Esophageal Neoplasms, Smoking, Alcohol Drinking, the JACC Study, Cohort Studies

## Abstract

BACKGROUND: Using a large-scale cohort of about 110,000 people established in 45 areas throughout Japan from 1988 through 1990, the study attempted to uncover the joint effects of combined smoking and alcohol intake on esophageal cancer mortality.

METHODS: A cohort established from 1988 through 1990 included 46,465 men and 64,327 women aged 40 years and older and younger than 80. The number of female smokers and drinkers was low, and women were excluded from the analysis for that reason. In addition, 308 people with histories of malignant neoplasm, and 3,579 with unclear smoking and drinking data were also excluded, resulting in 42,578 people available for analysis. A follow-up of these individuals was conducted until 1999. Cox proportional hazards model was used for the analysis.

RESULTS: The joint effects of number of cigarettes and amount of alcohol consumed per day were compared with non-smokers and non-drinkers or those consuming less than one unit of alcohol per day. An increased synergistic esophageal cancer mortality risk (3.88) for both smoking and drinking was observed for those smoking 20 cigarettes or less per day and drinking one unit of alcohol or more but less than three units per day, with the risk rising (6.30) for those smoking at least 21 cigarettes and drinking at least three units of alcohol per day. Even in non-smokers with increased alcohol consumption, and in non-drinkers or those drinking at most one drink per day with increased smoking, no increased risk was observed.

CONCLUSIONS: In this cohort study of a Japanese population, increased esophageal cancer mortality risk was observed only when both factors of alcohol and tobacco intake were present simultaneously.

The worldwide death rate from esophageal cancer, according to World Health Organization statistics from the year 2000, for men was 226,000, the fifth leading cause of death for that group, behind lung, gastric, liver, and colon cancers. For women, the number of deaths totaled 110,000, the 7th leading cause of death behind breast, lung, gastric, colon, uterine, and ovarian cancers.^1^ In Japan, according to estimates from the 2001 vital statistics, 9026 men and 1651 women died of the disease, a continued increase in terms of number of deaths but unchanged in terms of age-adjusted mortality rate.^2^ According to results from the National Cancer Center’s Central Hospital, the five-year survival rate from 1992 through 1996 for esophageal cancer for men was 40%, indicating that this malignant neoplasm has poorer prognosis than the 72% for both gastric and colon cancers, and near the rate of 35% for lung cancer.^3^

Smoking and alcohol drinking as risk factors for esophageal cancer have been clarified by many case-control studies^4^^-^^9^ and three cohort studies.^10^^-^^12^ In all such studies, a consistent association was observed with smoking and alcohol intake both in Japan and in other countries, and therefore, that contribution is thought to be real. However, though there have been case-control studies on how smoking and alcohol intake interact to contribute to esophageal cancer,^8^^,^^9^ no detailed cohort-study analysis has been conducted. One reason for this is the need for a long-term follow-up of an extensive cohort. This study’s objective was to elucidate which characteristics of smoking and alcohol intake contribute to esophageal cancer mortality, using data from the Japan Collaborative Cohort Study (JACC Study) for Evaluation of Cancer Risk sponsored by the Ministry of Education, Science, Sports and Culture of Japan (Monbusho). Another purpose was to clarify the joint effects of smoking and alcohol intake.

## METHODS

### Study Population

Details of the cohort and follow-up procedures have been described elsewhere.^13^,^14^ In brief, a baseline survey was conducted in 45 areas throughout Japan from 1988 through 1990 by investigators from 24 centers. At the end of 1990, a total of 125,760 inhabitants were enrolled in this cohort. Among them, 110,792 subjects (46,465 men and 64,327 women; aged between 40 and 79 years at baseline) were followed up through the end of 1999. Because women who had smoked or drunk alcohol were insufficient in number, we excluded women from the analysis. For this study, 308 men were eliminated because they had a history of cancer; 3579 men were excluded because they did not give information about their smoking or drinking status. The 42,578 men remaining were available for analysis. The research protocol was approved by the Ethical Boards of Nagoya University School of Medicine and Wakayama Medical University.

### Baseline Data Collection

The baseline data were collected utilizing a self-administered questionnaire, which included details about alcohol consumption, smoking history, dietary habits, health conditions, healthy habits, exercise, occupation, educational background, and subjective view of life. Smoking habit was established by asking the subjects whether they were a non-, ex-, or current smoker. Those who were current smokers were asked about the amount of cigarettes consumed per day and age at which smoking started. Packs were calculated by the number of cigarettes smoked divided by 20, and pack-years were calculated as the product of packs per day and the duration of smoking. Alcohol intake was based on the usual yearly intake of *sake* (Japanese rice wine), *shochu* (Japanese spirits), beer, whisky, and wine among current drinkers. The daily amount of alcohol consumption was assessed in terms of the conventional alcohol unit (*go*) of Japanese *sake*, one unit of which is equivalent to about 22 grams of alcohol.

### Follow-up Procedure

The date and cause of death were annually or biannually confirmed, with the permission of the director-general of the Prime Minister’s Office (Ministry of Public Management, Home Affairs, Post and Telecommunications). The date of move-out from the study area was also annually verified by the investigator in each area by reviewing population-register sheets of the cohort members. For deceased subjects, the causes of death were identified with underlying causes of death by reviewing all death certificates in each area once a year with permission from the Director-General of the Prime Minister’s Office. The underlying causes of death were coded according to the International Statistical Classification of Diseases, Injuries, and Causes of Death, Ninth Revision (ICD-9),^15^ from baseline through 1994, and the International Statistical Classification of Diseases and Related Health Problems, Tenth Revision (ICD-10),^16^ in and after 1995. Death from esophageal cancer was determined by the coding 150.0 through 150.9 for ICD-9 and C15.0 through C15.9 for ICD-10.

### Statistical Analysis

Cox proportional hazards model^17^ was used to estimate the relative risk due to cigarette smoking or alcohol intake adjusted by age and study centers. To test significance, the two-sided Wald’s test was used. All calculations were performed with Statistical Analysis System^®^ (SAS) software.^18^

## RESULTS

Age composition and smoking and alcohol intake status at the time of baseline study are shown in Table 1. Subjects in their 40s, 50s, and 60s of age comprised about 30% of the total, with those in their 70s making up about 10%. The number of current smokers made up more than half the total, whereas ex-smokers comprised 25%. Current drinkers comprised 75% of the total, while ex-drinkers made up only 6%.

**Table 1.** Baseline characteristics of study participants (men).

Factors	Categories	No.	(%)
Total		42,578	100.0

Age at baseline (year)	40-49	11,265	26.5
	50-59	12,941	30.4
	60-69	12,605	29.6
	70-79	5,767	13.5

Tobacco	Non-smokers	8,743	20.5
	Ex-smokers	11,155	26.2
	Smokers	22,680	53.3

Alcohol	Non-drinkers	8,010	18.8
	Ex-drinkers	2,629	6.2
	Drinkers	31,939	75.0

Characteristics of the smokers are shown in Table 2. With respect to age at the start of smoking, those starting between the ages of 20 to 24 years comprised about 60% of the total, while those 25 years of age or older and those between the ages of 10 and 19 made up slightly less than 20%, each. As for number of cigarettes smoked daily, those smoking from 11 to 20 comprised 56% of the total, those smoking 21 to 30 cigarettes made up 17%, and those smoking from 1 to 10 comprised 16%. Subjects smoking 31 or more cigarettes per day comprised only 9% of the total. With respect to number of smoking years, the largest category comprised those who smoked from 35.1 to 45.0 years (32% of the total). By pack-years, the category of 40 or more comprised 33% of the total, 30 to 39 made up 23%, and 20 to 29 comprised 24%.

**Table 2.** Characteristics of current smokers (men).

Categories	No.	(%)
Total	22,680	100.0

Age at baseline (year)		
25+	3,768	16.6
20-24	13,503	59.5
10-19	4,254	18.8
Unknown	1,155	5.1

Cigarettes smoked per day		
1-10 cigarettes/day	3,618	16.0
11-20	12,674	55.9
21-30	3,944	17.4
31+	2,141	9.4
Unknown	303	1.3

Duration of smoking (year)		
-25	4,851	21.4
25.1-35.0	6,232	27.5
35.1-45.0	7,286	32.1
45.1-	3,170	14.0
Unknown	1,141	5.0

Cumulative amount of smoking		
1-19.9 pack-years	3,234	14.3
20.0-29.9	5,404	23.8
30.0-39.9	5,196	22.9
40.0+	7,567	33.4
Unknown	1,279	5.6

Characteristics of the drinkers are shown in Table 3. In terms of amount of alcohol consumed per day, those drinking 1.0 to 1.9 units comprised the largest group (32% of the total), with those drinking 2.0 to 2.9 units making up the next largest group (27%). Those who drank 3.0 units or more made up 13% of the total, and those drinking less than 1.0 unit only comprised 7% of the total. As for years of alcohol intake, the largest group was those drinking 25 years or less (35% of the total), followed by those drinking alcohol 25.1 to 35.0 years (21%), and those drinking 35.1 to 45.0 years (15%). By unit-year, 40 or more was the largest group (37% of the total), followed by the group of less than 30 (23%). In terms of type of alcohol, those drinking sake comprised the largest group (56% of the total), with the next largest group making up those drinking beer (40%), followed by shochu (15%), and then whiskey (14%). Wine drinkers comprised only 5% of the total.

**Table 3.** Characteristics of alcohol drinkers (men).

Categories	No.	(%)
Total	31,939	100.0

Alcohol units* consumed per day		
<1.0 units/day	2,273	7.1
1.0-1.9	10,346	32.4
2.0-2.9	8,587	26.9
3.0+	4,049	12.7
Unknown	6,684	20.9

Duration of alcohol drinking (year)		
-25.0	11,298	35.4
25.1-35.0	6,780	21.2
35.1-45.0	4,683	14.7
45.1+	1,836	5.8
Unknown	7,342	23.0

Cumulative amount of alcohol intake (unit-year)		
1-29.9	7,493	23.5
30.0-39.9	3,007	9.4
40.0+	11,970	37.5
Unknown	9,469	29.7

Type of alcohol (multiple answer)		
Sake	17,868	55.9
Shochu^†^	4,895	15.3
Beer	12,927	40.5
Whisky	4,518	14.1
Wine	1,491	4.7

* : One unit contains about 22g of alcohol.

† : Japanese spirits.

Hazard ratio of esophageal cancer by smoking status is shown in Table 4, after adjusting for age and study center. Using non-smokers as the standard, current smokers and ex-smokers had a significantly higher mortality risk (4.36 and 2.71, respectively). Considering age when smokers started smoking, in all categories, the risk rose three to five times, but no dose-response association was observed. This was also the case when looking at the data by number of cigarettes smoked per day. With respect to number of smoking years, a higher risk trend was observed as number of smoking years increased. With regard to investigation of pack-year, however, no dose-response relationship was observed.

**Table 4.** Hazard Ratio (HR) of death from esophageal cancer according to smoking status at baseline (men).

Variables	Person-years	No. of deaths	HR* (95% confidence interval)	P for trend
Non-smokers	86,446	7	1.00 (reference)	
Ex-smokers	107,708	25	2.71 (1.16-6.36)	
Smokers	220,492	68	4.36 (2.00-9.52)	

Age at start of smoking				
Non-smokers	86,446	7	1.00 (reference)	
25+	36,321	13	3.85 (1.54-9.64)	
20-24	130,874	38	4.89 (1.98-12.07)	
10-19	41,885	13	3.24 (1.06-9.89)	0.391

Cigarettes smoked per day				
Non-smokers	86,446	7	1.00 (reference)	
1-10 cigarettes/day	34,185	15	5.11 (2.07-12.65)	
11-20	123,009	39	4.42 (1.97-9.92)	
21-30	39,206	8	3.19 (1.11-9.19)	
31+	21,177	5	4.33 (1.25-14.99)	0.431

Years of smoking				
Non-smokers	86,446	7	1.00 (reference)	
-25.0	49,108	4	2.05 (0.42-9.98)	
25.1-35.0	62,652	13	3.54 (1.27-9.89)	
35.1-45.0	70,674	32	5.34 (2.32-12.30)	
45.1+	26,774	15	4.85 (1.62-14.53)	0.014

Cumulative amount of smoking				
Non-smokers	86,446	7	1.00 (reference)	
1-19.9 pack-years	31,784	6	3.24 (1.06-9.89)	
20.0-29.9	53,136	16	4.89 (1.98-12.07)	
30.0-39.9	51,039	14	3.85 (1.54-9.64)	
40.0+	71,896	28	4.86 (2.11-11.21)	0.086

*Hazard ratio adjusted for age and centers.

Hazard ratio of esophageal cancer based on drinking status is shown in Table 5. Current drinkers had a significantly increased risk (2.40) compared with non-drinkers. Ex-drinkers had a 2.43 times higher risk, but the risk was not considered significant. By amount of alcohol consumed per day, esophageal cancer risk increased with increased alcohol intake. As for number of years of alcohol intake, no dose-response relationship was observed. By unit-year, the group of subjects with 40 unit-years or greater had the highest risk, but that risk was insignificant in a test of linear trend at a p value of 0.05. Looking at the data by type of alcohol consumed, the highest risk was with wine (6.24), followed by *shochu* (3.40), and *sake* (2.72). For beer and whiskey no significant increase in risk was observed.

**Table 5.** Relative risk of death from esophageal cancer according to alcohol intake status at baseline (men).

Variables	Person-years	No. of deaths	HR* (95% confidence interval)	P for trend
Non-drinkers	76,521	9	1.00 (reference)	
Ex-drinkers	22,754	8	2.43 (0.91-6.47)	
Drinkers	315,370	83	2.40 (1.20-4.80)	

Alcohol units^†^ consumed per day				
Non-drinkers	76,521	9	1.00 (reference)	
<1.0 units/day	21,532	2	1.47 (0.28-7.68)	
1.0-1.9	99,786	16	1.58 (0.65-3.86)	
2.0-2.9	83,124	31	3.74 (1.62-8.66)	
3.0+	39,027	18	6.39 (2.54-16.12)	0.028

Years of alcohol drinking				
Non-drinkers	76,521	9	1.00 (reference)	
-25.0	111,842	14	1.71 (0.64-4.60)	
25.1-35.0	66,150	19	3.23 (1.32-7.92)	
35.1-45.0	43,855	18	3.23 (1.33-7.81)	
45.1+	15,706	7	2.77 (0.85-9.03)	0.100

Cumulative amount of alcohol intake				
Non-drinkers	76,521	9	1.00 (reference)	
1-29.9 unit-years	73,262	4	0.68 (0.19-2.42)	
30.0-39.9	29,201	6	2.31 (0.75-7.06)	
40.0+	113,822	46	3.80 (1.70-8.46)	0.089

Type of alcohol				
Non-drinkers	76,521	9	1.00 (reference)	
Sake	172,587	48	2.72 (1.22-6.08)	
Shochu^‡^	47,499	15	3.40 (1.33-8.68)	
Beer	125,265	17	1.42 (0.58-3.52)	
Whisky	44,414	9	2.60 (0.91-7.41)	
Wine	14,956	6	6.24 (1.53-25.37)	

* : Hazard ratio adjusted for age and centers.

† : One unit contains about 22g of alcohol.

‡ : Japanese spirits.

Results of investigation into the joint effects of smoking and drinking are shown in Table 6. Dividing smokers into the categories non-smoker, ex-smoker, and smoker, and drinkers into non-drinker, ex-drinker, and drinker, smokers and drinkers were compared with non-smokers and non-drinkers. The comparison showed that the hazard ratio by point estimate for the former group increased to 2.37, not significant at a p-value of 0.05. In terms of joint effects based on number of cigarettes and amount of alcohol consumed per day, comparing the non-smokers and non-drinkers or those drinking less than one unit per day with those smoking less than 20 cigarettes per day and drinking one or more units of alcohol but less than three, the hazard ratio was 3.88, which increased to 6.30 for those smoking 21 cigarettes or more and drinking three units or more per day. These results indicated a synergistic increase in risk for combined smoking and drinking. However, for non-smokers even with increased alcohol consumption, and for non-drinkers and those drinking less than one unit with increased cigarette intake, no increase in risk was observed. Looking at the cumulative effects of smoking and drinking using as a standard the group of non-smokers and non-drinkers or those with 30 unit-years, the hazard ratio was 5.78 for smokers with less than 40 pack-years and for drinkers with 40 unit-years or more. For smokers with 40 pack-years or more and alcohol drinkers with 40 unit-years or more, an increased risk of 7.01 was observed. In non-smokers, however, even with increased cumulative alcohol intake, and in non-drinkers or in those with less than 30.0 unit-years even with increased cumulative cigarettes smoked, no increased esophageal cancer mortality risk was observed.

**Table 6.** Joint effects of smoking and alcohol consumption on risk of esophageal cancer death (men).

Tobacco	Alcohol	No. of deaths	HR* (95% confidence interval)
By smoking and drinking status			
Non-smokers	Non-drinkers	4	1.00 (reference)
Non-smokers	Ex-drinkers	1	1.10 (0.12-10.24)
Non-smokers	Drinkers	2	0.18 (0.03-1.02)
Ex-smokers	Non-drinkers	1	0.34 (0.04-3.12)
Ex-smokers	Ex-drinkers	3	1.47 (0.31-7.08)
Ex-smokers	Drinkers	21	1.39 (0.47-4.10)
Smokers	Non-drinkers	4	0.74 (0.18-3.02)
Smokers	Ex-drinkers	4	2.19 (0.51-9.40)
Smokers	Drinkers	60	2.37 (0.85-6.58)

By smoked cigarettes and consumed alcohol in units^†^ per day			
Non-smokers	Non- or <1.0 units/day drinkers	4	1.00 (reference)
Non-smokers	1.0-2.9	0	-
Non-smokers	3.0+	0	-
1-20 cigarettes/day	Non- or <1.0	3	0.81 (0.18-3.73)
1-20	1.0-2.9	35	3.88 (1.19-12.69)
1-20	3.0+	6	4.01 (0.93-17.31)
21+	Non- or <1.0	0	-
21+	1.0-2.9	4	1.80 (0.37-8.78)
21+	3.0+	6	6.30 (1.33-29.76)

By cumulative amount of smoking and alcohol intake			
Non-smokers	Non- or <30.0 unit-year drinkers	4	1.00 (reference)
Non-smokers	30.0-39.9	0	-
Non-smokers	40.0+	0	-
1-39.9 pack-years	Non- or <30.0	2	0.65 (0.12-3.70)
1-39.9	30.0-39.9	3	4.56 (0.88-23.64)
1-39.9	40.0+	21	5.78 (1.71-19.55)
40.0+	Non- or <30.0	2	0.89 (0.16-4.96)
40.0+	30.0-39.9	2	12.33 (1.86-81.64)
40.0+	40.0+	15	7.01 (2.01-24.45)

* : Hazard ratio adjusted for age and centers.

† : One unit contains about 22g of alcohol.

## DISCUSSION

For the 42,578 males, a follow-up was conducted over a period of about 10 years, analyzing the joint effects of smoking and drinking on esophageal cancer mortality. It was found from the follow-up that combined smoking and drinking, established previously as risk factors for esophageal cancer, were also clearly shown to contribute to the mortality in this cohort. In this study with regard to smoking, a trend of greater esophageal cancer mortality risk was observed the longer the duration of smoking, but no dose-response was observed for age at start of smoking, number of cigarettes smoked daily, or cumulative amount of cigarettes smoked. For alcohol intake, a dose-response association was observed for amount of alcohol consumed per day, but for number of drinking years or cumulative amount of alcohol intake, no dose-response association was observed. In a previous case-control study, for smoking, length of smoking period was most strongly associated, whereas for alcohol intake, average amount of alcohol consumption was most strongly associated, information that conform with the results obtained in this study.^19^ However, this study did not observe any association with cumulative amount of cigarette consumption or cumulative alcohol intake. The reason for this disparity is possibly because, compared with France, alcohol consumed in Japan is largely beer, which has low alcohol content, with a low level of wine intake. Regarding cumulative amount of smoking, relatively low consumption of high alcohol content drinks may have made any significant association hard to detect.

By type of alcohol, Japan is characterized by widespread consumption of *sake*, an alcohol unique to Japan. In terms of esophageal cancer, no association was observed with low alcohol content beer, but the risk rose with wine, *shochu*, *sake*, and whiskey, in that order. Wine had the strongest association. However, whether this was because wine drinkers were more likely to contract esophageal cancer than *sake* drinkers, or whether the risk increased not because wine drinkers drank wine but because they consumed more per day is an issue that requires future investigation. The limitation of this study is high percentage of unknown categories among drinkers. This might cause an information bias.

With respect to the interaction of smoking and drinking, a synergistic effect was proven in case-control studies conducted to date,^8^^,^^9^ but such effect has not yet been proven in a cohort study. Through analysis of the relationship, we investigated whether smoking or alcohol intake contributed to the onset of esophageal cancer independently or whether they are a risk only when present simultaneously. For non-smokers, no esophageal cancer mortality risk was observed even in the smokers who consumed the largest amount of alcohol. Among non-drinkers and those who consumed less than one unit of alcohol per day, no increased risk was observed even when daily tobacco consumption increased from 20 cigarettes or less to 21 cigarettes or more. As for cumulative effects among non-drinkers and drinkers with less than 30 unit-years, no increased risk was observed even when cumulative pack-years of less than 40 increased to 40 or more. A synergetic effect was observed in both analyses by amount per day and cumulative amount, but not in the analysis by smoking and drinking status. These findings imply that amounts of smoking and drinking are important to increase esophageal cancer.

These results suggest that simultaneous exposure to both factors is more important than independent exposure to either smoking or drinking in terms of the occurrence of esophageal cancer in Japan. The results show that quitting smoking, curtailing alcohol intake, and reducing the consumption of high alcohol content drinks can be expected to greatly reduce the current rate of esophageal cancer occurrence.

## MEMBER LIST OF THE JACC STUDY GROUP

The present investigators involved, with the co-authorship of this paper, in the JACC Study and their affiliations are as follows: Dr. Akiko Tamakoshi (present chairman of the study group), Nagoya University Graduate School of Medicine; Dr. Mitsuru Mori, Sapporo Medical University School of Medicine; Dr. Yutaka Motohashi, Akita University School of Medicine; Dr. Ichiro Tsuji, Tohoku University Graduate School of Medicine; Dr. Yosikazu Nakamura, Jichi Medical School; Dr. Hiroyasu Iso, Institute of Community Medicine, University of Tsukuba; Dr. Haruo Mikami, Chiba Cancer Center; Dr. Yutaka Inaba, Juntendo University School of Medicine; Dr. Yoshiharu Hoshiyama, University of Human Arts and Sciences; Dr. Hiroshi Suzuki, Niigata University School of Medicine; Dr. Hiroyuki Shimizu, Gifu University School of Medicine; Dr. Hideaki Toyoshima, Nagoya University Graduate School of Medicine; Dr. Kenji Wakai, Aichi Cancer Center Research Institute; Dr. Shinkan Tokudome, Nagoya City University Graduate School of Medical Sciences; Dr. Yoshinori Ito, Fujita Health University School of Health Sciences; Dr. Shuji Hashimoto, Fujita Health University School of Medicine; Dr. Shogo Kikuchi, Aichi Medical University School of Medicine; Dr. Akio Koizumi, Graduate School of Medicine and Faculty of Medicine, Kyoto University; Dr. Takashi Kawamura, Kyoto University Center for Student Health; Dr. Yoshiyuki Watanabe, Kyoto Prefectural University of Medicine Graduate School of Medical Science; Dr. Tsuneharu Miki, Graduate School of Medical Science, Kyoto Prefectural University of Medicine; Dr. Chigusa Date, Faculty of Human Environmental Sciences, Mukogawa Women’s University ; Dr. Kiyomi Sakata, Wakayama Medical University; Dr. Takayuki Nose, Tottori University Faculty of Medicine; Dr. Norihiko Hayakawa, Research Institute for Radiation Biology and Medicine, Hiroshima University; Dr. Takesumi Yoshimura, Fukuoka Institute of Health and Environmental Sciences; Dr. Akira Shibata, Kurume University School of Medicine; Dr. Naoyuki Okamoto, Kanagawa Cancer Center; Dr. Hideo Shio, Moriyama Municipal Hospital; Dr. Yoshiyuki Ohno, Asahi Rosai Hospital; Dr. Tomoyuki Kitagawa, Cancer Institute of the Japanese Foundation for Cancer Research; Dr. Toshio Kuroki, Gifu University; and Dr. Kazuo Tajima, Aichi Cancer Center Research Institute.
